# Vorinostat differentially alters 3D nuclear structure of cancer and non-cancerous esophageal cells

**DOI:** 10.1038/srep30593

**Published:** 2016-08-09

**Authors:** Vivek Nandakumar, Nanna Hansen, Honor L. Glenn, Jessica H. Han, Stephanie Helland, Kathryn Hernandez, Patti Senechal, Roger H. Johnson, Kimberly J. Bussey, Deirdre R. Meldrum

**Affiliations:** 1 School of Electrical, Computer and Energy Engineering, Arizona State University, Tempe, Arizona; 2 Center for Biosignatures Discovery Automation, Biodesign Institute, Tempe, Arizona

## Abstract

The histone deacetylase (HDAC) inhibitor vorinostat has received significant attention in recent years as an ‘epigenetic’ drug used to treat solid tumors. However, its mechanisms of action are not entirely understood, particularly with regard to its interaction with the aberrations in 3D nuclear structure that accompany neoplastic progression. We investigated the impact of vorinostat on human esophageal epithelial cell lines derived from normal, metaplastic (pre-cancerous), and malignant tissue. Using a combination of novel optical computed tomography (CT)-based quantitative 3D absorption microscopy and conventional confocal fluorescence microscopy, we show that subjecting malignant cells to vorinostat preferentially alters their 3D nuclear architecture relative to non-cancerous cells. Optical CT (cell CT) imaging of fixed single cells showed that drug-treated cancer cells exhibit significant alterations in nuclear morphometry. Confocal microscopy revealed that vorinostat caused changes in the distribution of H3K9ac-marked euchromatin and H3K9me3-marked constitutive heterochromatin. Additionally, 3D immuno-FISH showed that drug-induced expression of the DNA repair gene MGMT was accompanied by spatial relocation toward the center of the nucleus in the nuclei of metaplastic but not in non-neoplastic cells. Our data suggest that vorinostat’s differential modulation of 3D nuclear architecture in normal and abnormal cells could play a functional role in its anti-cancer action.

The aberrant expression of histone deacetylase (HDAC) enzymes in many epithelial cancers, including those of the lung, breast, gastrointestinal tract, prostate and ovaries^1^,^2^,^3^,^4^, has stimulated interest in the potential utility of HDAC inhibitors (HDACi) as therapeutics. Vorinostat is the first HDAC inhibitor approved by the Food and Drug Administration (FDA) to treat advanced cutaneous T-cell lymphoma (CTCL)^5^,^6^. It is known to inhibit the activity of class I and class II HDAC enzymes and is the focus of multiple clinical trials as a potential mono- or combination-drug therapy for solid tumors^7^. Extensive studies demonstrate vorinostat acts through multiple, complex anticancer mechanisms. In addition to engendering histone acetylation leading to alterations in gene expression, vorinostat inhibits proliferation, induces differentiation, causes cell-cycle arrest, results in double-strand breaks at micromolar concentrations, and triggers both apoptosis and autophagy in cancer cells^8^,^9^,^10^,^11^. Mechanistic studies implicate numerous non-histone proteins, including the STAT and Bcl-2 protein families, HSP90, β-catenin, and HIF1-α, as essential factors in the drug’s action. However, it is not clear how acetylation-induced chromatin structural rearrangement contributes to vorinostat’s mechanism of action.

Chromatin is thought to be spatially organized into higher-order structures that ultimately exhibit a non-random three-dimensional (3D) organization within cell nuclei^12^. The 3D genome modulates nuclear shape through its binding with proteins in the nuclear envelope^13^. The 3D spatial organization of the genome also plays a role in the epigenetic control of gene expression^14^,^15^,^16^,^17^,^18^. Advances in fluorescence microscopy and image analysis have enabled the identification of specific patterns in the organization of genomic regions for different cancers^19^,^20^,^21^. These analytical capabilities have facilitated closer correlations between cytological-scale aberrations, such as nuclear shape and size, and higher-order chromatin organization. Innovations in single-cell optical computed tomography of fixed cells enable quantitative isotropic absorption measurements in 3D^22^. This is clinically relevant since it relates underlying chromatin restructuring with the parameters traditionally used qualitatively by pathologists to diagnose malignancy based on staining with absorption dyes such as hematoxylin and eosin (H&E)^23^.

Little is known about how vorinostat influences the 3D nuclear architecture of cells as they progress from normal to pre-cancer to cancer, and whether or how malignancy-associated changes in nuclear architecture could modulate the drug’s cancer-specific pharmacological effects. Several prior studies applied fluorescence microscopy methods to report cytological-scale chromatin decondensation in epithelial cells upon treatment with the HDAC inhibitors trichostatin A or sodium butyrate^24^,^25^,^26^,^27^. Kortenhorst *et al*. have used 2D absorption-mode microscopy to show that treatment with the HDAC inhibitor valproic acid causes dose dependent alterations in the 2D nuclear structure of prostate cancer cells^28^. Alterations of gene expression by vorinostat correlate with site-specific accumulation of acetylated histones^29^, and the altered histone modification patterns are typically assumed to result in the higher-order structural reorganization of the corresponding genomic loci. However, this has not been explicitly shown. Further, a comparative analysis of the effects of clinically relevant doses of vorinostat on the 3D nuclear architecture of cancerous epithelial cells relative to both normal and pre-cancerous epithelial cells has not been reported. Thus, the extent and pattern of chromatin reorganization along a neoplastic progression spectrum is not known.

We applied a combination of novel and traditional quantitative microscopy methods to pursue these questions. First, we used high-resolution single-cell optical computed tomography (cell CT) imaging^30^ of fixed cells and automated morphometric analysis to accurately quantify the impact of clinically-relevant doses of vorinostat on 3D nuclear structure of hTERT-immortalized squamous (henceforth referred to as “normal”), pre-cancerous, and malignant esophageal epithelial cell lines. Single-cell optical CT yields images that have sub-micron isotropic resolution (i.e. the same spatial resolution along all three axes). Cell CT generates a spherical point spread function (PSF) that enables an orientation-independent quantification of morphology in all three spatial dimensions, which is not accessible via traditional imaging modes. Second, we applied confocal microscopy-based quantitative 3D immuno-FISH to assess whether and how malignancy impacted the interplay between drug-induced changes in 3D nuclear architecture and gene expression. We report significant differences between the 3D nuclear structure patterns of malignant and non-malignant cells upon exposure to the same dose of vorinostat. Drug-treated cancer cells exhibited a pan-interphase decrease in the magnitude and heterogeneity of diagnostically important morphological parameters such as nuclear size (volume), nuclear-cytoplasmic ratio, and chromatin clumpiness relative to pre-cancerous and normal cells. Additionally, vorinostat-induced expression of the DNA repair gene MGMT was accompanied by an increase in its co-localization with H3K9ac-marked euchromatin domains in pre-cancerous and malignant cells, but not normal cells, and an inward spatial relocation of the gene locus in pre-cancerous cells. Our results highlight the utility of quantifying 3D nuclear architecture to monitor cellular response to vorinostat, and provide evidence to support a functional role for nuclear architecture in vorinostat’s anti-cancer mechanism.

## Results

### Vorinostat reduces viability of esophageal adenocarcinoma cells relative to metaplastic and normal squamous epithelial cells

**Figure 1:**
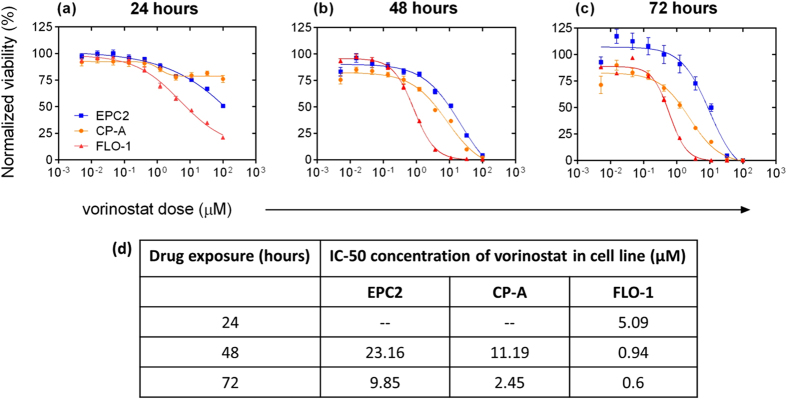
Vorinostat reduces viability of FLO-1 esophageal adenocarcinoma cells relative to metaplastic CP-A and normal squamous EPC2 cells. Drug-dose response (DDR) curves after: (**a**) 24, (**b**) 48 and (**c**) 72 hours of vorinostat exposure. (**d**) FLO-1 cells have a significantly lower IC-50 values compared to CP-A and EPC2. The missing IC50 values (depicted as --) for EPC2 and CP-A cells at the 24 hour time point in (**d**) indicate that these cells had minimal response to the drug at this time point. The resulting data (n = 6 for each dose) were analyzed by using the Dose-response-inhibition, variable slope function in Prism 6 (Graphpad Software, San Diego, CA) after normalization to growth of cells alone and with the vehicle control DMSO. Error bars represent the standard error of the mean (SEM).

To assess the inhibitory effect of vorinostat on esophageal adenocarcinoma, we measured the sensitivity of normal squamous (EPC2), metaplastic Barrett’s (CP-A), and esophageal adenocarcinoma (FLO-1) cell lines to increasing concentrations (100 nM to 1 mM) of vorinostat across three time points: 24, 48, and 72 hours. Drug-dose-response (DDR) curves for all three time points (Fig. 1(a–c)) and the corresponding IC50 concentrations (Fig. 1(d)) revealed the FLO-1 cancer cells to be consistently most sensitive (i.e. had the lowest IC50 concentration) to the drug compared to metaplastic CP-A and normal squamous EPC2 cells. We chose the 48 hour time point for our subsequent experiments because it was the shortest time that gave a sigmoidal-shaped curve for FLO-1. At this time point, the IC50 concentration of vorinostat required for FLO-1 cells (0.94 μM) was 11 times lower than that of CP-A cells (11.19 μM) and 24 times lower than EPC2 cells (23.16 μM).

### Vorinostat treatment preferentially affects the 3D nuclear structure of adenocarcinoma cells relative to metaplastic and normal cells in interphase

As a first step to assess the impact of vorinostat on 3D interphase nuclear architecture of normal and abnormal cells, we applied single-cell optical computed tomography (CT) imaging and custom 3D automated image analysis methods to accurately quantify the 3D nuclear structure of fixed, hematoxylin-stained cells, and assess morphological heterogeneity in vorinostat-treated cells relative to vehicle control (DMSO). Mimicking a clinically relevant treatment scenario, we performed our experiment on unsynchronized, interphase EPC2, CP-A, and FLO-1 cells subjected to the IC50 drug concentration for FLO-1 cancer cells. Additionally, we used hematoxylin-stained cells and evaluated the following diagnostically relevant morphological parameters that can be correlated with clinical pathology: nuclear size (volume in cubic microns), nuclear to cytoplasmic (NC) ratio, nuclear shape convexity (to measure the irregularity in nuclear shape), and the number of dense intra-nuclear clumps (to reflect chromatin texture). The formulae used to quantitatively measure these parameters in volumetric optical CT images are defined in Supplementary Table S1.

**Figure 2:**
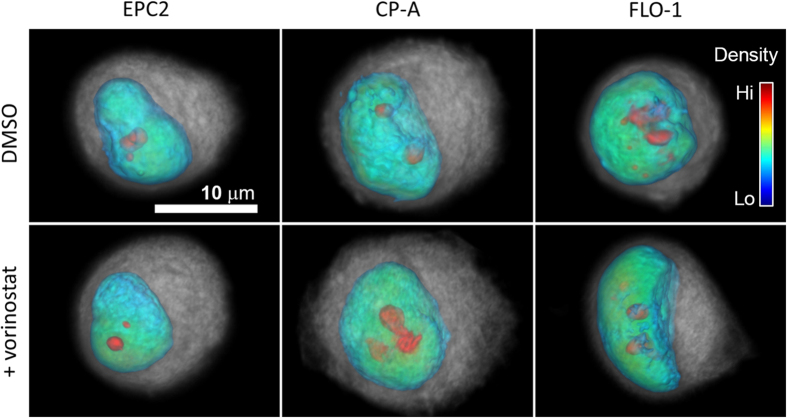
Treatment with vorinostat preferentially decreases the nuclear size and intra-nuclear clumpiness in FLO-1 cancer cells relative to metaplastic and normal squamous esophageal epithelial cells. Representative volume renderings of cells imaged by single-cell optical CT illustrate the surface morphology and nuclear interior of DMSO-treated (top row), and vorinostat-treated (bottom row) EPC2 (left column), CP-A (middle column), and FLO-1 (right column) cells. The cytoplasm is gray, nuclear envelope is blue, and increasing nuclear density is color coded from green to red as indicated by the color scale bar.

**Figure 3:**
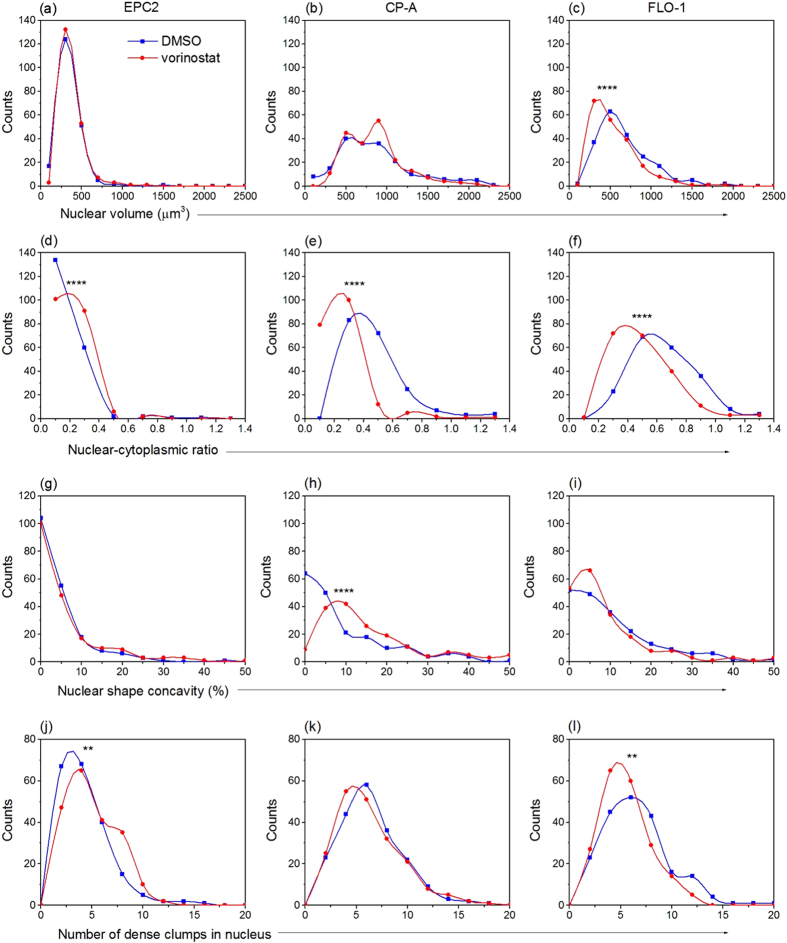
Vorinostat treatment differentially alters 3D nuclear structure and heterogeneity in normal squamous (EPC2), metaplastic Barrett’s (CP-A), and adenocarcinoma (FLO-1) esophageal epithelial cells. Histograms illustrate the impact of 0.94 μM vorinostat (red curves) on: (**a**–**c**) nuclear volume; (**d**–**f**) nuclear cytoplasmic (NC) ratio; (**g**–**i**) nuclear shape concavity; (**j-l**) the number of intra-nuclear clumps and morphological heterogeneity relative to DMSO treated cells (blue curves). P-value indicators: **p < 0.01, ****p < 0.0001. All three cell types exhibited statistically significant changes in NC ratio upon treatment; NC ratio increased in EPC2 cells and decreased in CP-A and FLO-1 cells. Treated FLO-1 cells additionally presented reductions in nuclear volume and intra-nuclear clumpiness. Drug exposure also caused CP-A nuclei to become significantly more concave-shaped and caused an increase in intra-nuclear clumpiness in EPC2 cell nuclei. Histograms were generated from 200 cells per condition. Data were analyzed in Prism6 using the Mann-Whitney test.

Three-dimensional morphological analysis of 200 cells per condition from each cell type revealed several interesting trends that indicated more prominent morphological transformations in drug-treated cancer (FLO-1) cells relative to metaplastic (CP-A) and normal squamous (EPC2) cells. Our results are collectively summarized by representative volume renderings (Fig. 2), histograms (Fig. 3), and statistics of the computed morphological parameters (Table 1).

We found the nuclear size of FLO-1 cells decreased significantly (from 672.7 ± 22.9 μm^3^ to 553.2 ± 20.5 μm^3^, p < 0.0001) after exposure to drug, whereas this parameter was unaffected in EPC2 and CP-A cells. However, all three cell types underwent statistically significant alterations in their NC ratios (p < 0.0001, Table 1) upon treatment. The NC ratio of drug-treated FLO-1 and CP-A cells decreased, and surprisingly, increased in normal EPC2 cells, suggesting a significant decrease in the cell volume of the latter. Change in 3D nuclear shape – as measured by the nuclear shape concavity index – did not appreciably change in drug-treated normal and cancer cells, but significantly increased (from 12.7 ± 1.3% to 27.1 ± 2.4%, p < 0.0001) in metaplastic cells. Exposure to the drug also caused a differential response in the intranuclear clumpiness of nuclear content (measured by the number of dense clumps within nuclei) between the three cell types. Clumpiness decreased (p = 0.0017, Table1) in cancer cells after treatment, was unaffected in metaplastic cells, and increased (p = 0.00063) in normal squamous cells.

We compared our cytological-scale observations of vorinostat-induced nuclear re-organization from optical CT images of gel-suspended cells with conventional confocal fluorescence microscopy imaging of adhered EPC2, CP-A, and FLO-1 cells immunolabeled for proteins associated with nuclear shape and higher-order chromatin structure. Specifically, we labeled lamin A/C to mark the nuclear lamina; the histone marks H3K9ac, H3K9me3, and H3K27me3 to visualize euchromatin, constitutive heterochromatin, and facultative heterochromatin respectively; and fibrillarin to identify nucleolar regions. We observed an increase in nuclear shape concavity and nuclear surface undulations in a subset of CP-A cells only, and not in EPC2 or FLO-1 cells (Supplementary Figure S1). Similarly, drug-treated FLO-1 cells exhibited an increase in the expression and diffused localization of H3K9ac, a prominent peripheral localization of H3K9me3, and a decrease in the number and size of fibrillarin-positive structures. H3K27me3 localization did not change appreciably relative to vehicle control, suggesting that it may not contribute to changes in clumpiness observed at the cytological scale. CP-A and EPC2 cell nuclei exhibited minimal changes in these chromatin protein patterns.

To assess whether the spatial localization patterns of these proteins correlated with a change in their expression upon drug treatment, we measured the total expression of the above immunolabeled protein targets by conventional bulk-cell based immunoblotting. As expected, we observed an increase in the expression of active histone marks H3K9ac (p < 0.0001) and H3K4me2 (p < 0.0001) only in drug-treated FLO-1 cells and not in the other cell types (Supplementary Figure S2). Interestingly, the expression levels of H3K9me3 and H3K27me3 did not change appreciably at the bulk-cell level (data not shown). Similarly, immunoblotting of lamin proteins revealed no changes in the expression of lamin A/C and lamin B2 in drug-treated FLO-1 cells. However, we observed a reduction in the expression of lamin B1 (p < 0.00001 compared to either CP-A or EPC2) in these cells, which could be tied to the reduction in nuclear size. Taken together, these results indicate that vorinostat-induced spatial reorganization of the genome may not have to do as much with changes in the expression levels of proteins implicated in genome architecture as it does with their altered spatial localization.

### Vorinostat-induced 3D nuclear structure alterations in adenocarcinoma cells are maintained in both G1 and G2 phases of cell cycle

**Figure 4:**
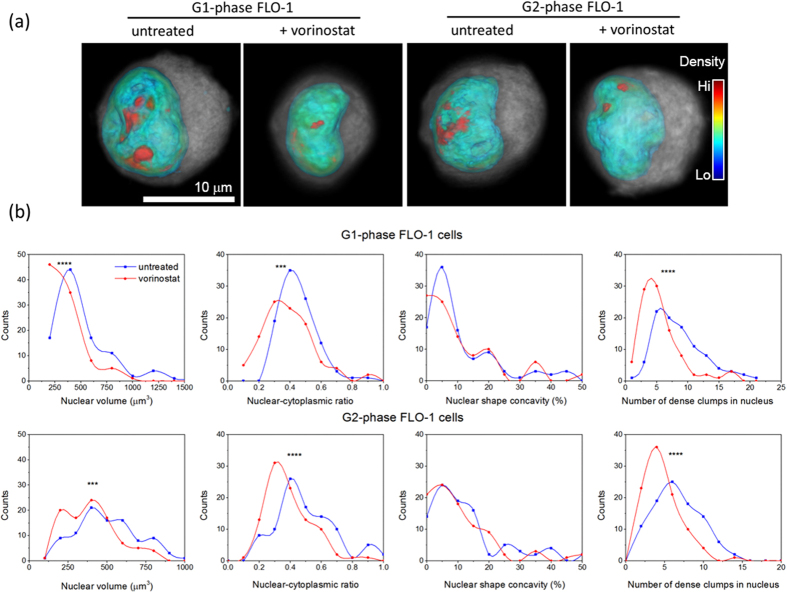
Vorinostat-induced 3D nuclear structure alterations in FLO-1 esophageal adenocarcinoma cells occur in both G1- and G2-phases of the cell cycle. (**a**) Representative pseudocolor volume renderings of cells imaged by single-cell optical CT illustrate a smoother surface morphology and nuclear interior in vorinostat-treated FLO-1 cells relative to untreated FLO-1 cells from the G1- and G2-phases of the cell cycle respectively. The cytoplasm is gray, nuclear membrane is blue, and increasing nuclear density is color coded from green to red as indicated by the color scale bar. (**b**) Histograms quantifying the morphological heterogeneity in nuclear size, nuclear cytoplasmic (NC) ratio, nuclear shape concavity, and number of intra-nuclear clumps in vorinostat-treated (red curves) relative to untreated cells (blue curves). Histograms were generated from 100 cells per condition. P-values are indicated by *: ***p < 0.001, ****p < 0.0001. Data were analyzed in Prism6 using the Mann-Whitney test.

We next checked whether the observed morphological effects of vorinostat on the nuclear structure of FLO-1 cancer cells were the consequence of cell cycle alterations induced by the drug. Fluorescence activated cell sorting (FACS) of Hoechst stained FLO-1 cells did not reveal any prominent cell cycle block or the induction of senescence upon treatment (Supplementary Figure S3, Supplementary Table S2) when compared to DMSO treated cells. We subsequently repeated our single-cell optical CT imaging and analysis procedure to assess the 3D nuclear structure of untreated and vorinostat-treated FLO-1 cell populations in the G1- and G2- phases of the cell cycle. Morphometric analysis of approximately 100 untreated and vorinostat-treated FLO-1 cancer cells revealed statistically significant decreases in the nuclear size, NC ratio, and intra-nuclear clumpiness of drug-treated cancer cells in both phases (Fig. 4 and Table 2). The drug-induced decrease in nuclear size was more pronounced in the G1 phase while the NC ratio was reduced to a greater degree in G2 compared to G1. Intra-nuclear clumpiness decreased to the same extent in both phases while global differences in nuclear shape were not significant. These results indicate that the reduction in nuclear size and clumpiness persist through interphase in FLO-1 cells.

### Vorinostat reduces morphological heterogeneity in adenocarcinoma cells

We used optical CT imaging to assess the impact of drug exposure on 3D nuclear morphological heterogeneity. A comparison of the histograms of the measured morphological parameters in the three cell types shown in Fig. 3 and the corresponding means and standard error in Table 1 revealed that drug-treated FLO-1 cancer cells have the most prominent reduction in heterogeneity of the affected parameters. Drug-treated metaplastic CP-A cells showed the next most prominent reductions in heterogeneity, while normal squamous EPC2 cells showed minimal change in heterogeneity upon drug treatment.

### Vorinostat-induced expression of DNA-repair gene MGMT is accompanied by differential repositioning of the gene in normal and abnormal esophageal epithelial cells

**Figure 5:**
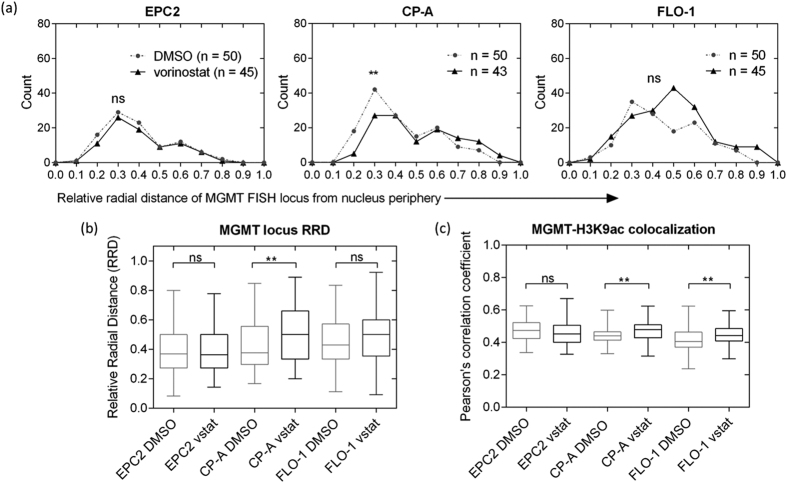
Increased MGMT expression after vorinostat exposure is accompanied by differential repositioning of gene locus and H3K9ac-colocalization between normal and abnormal esophageal epithelial cells. (**a**) Histograms illustrate the trends in nuclear positioning of the MGMT locus in DMSO (dotted gray)- and vorinostat (black)-treated normal EPC2, metaplastic CP-A, and malignant FLO-1 cells. Nuclear position was computed using the relative radial distance (RRD) metric (0 = periphery, 1 = center). Upon vorinostat exposure, the MGMT locus moved towards the nuclear interior in CP-A, but not in EPC2 or FLO-1 cells. P-value is indicated by *: ns = not significant (p > 0.05), **p < 0.01. Exposure to vorinostat in FLO-1 altered the distribution of RRDs such that they became normally distributed (A = 0.5933, p-value = 0.1198). Similar results were not seen in CP-A. (**b**) Box plots reflect the extent of heterogeneity in nuclear position of the MGMT locus in each cell type before and after vorinostat treatment. Treated FLO-1 cell nuclei exhibited the greatest heterogeneity in nuclear position of MGMT compared to CP-A and EPC2 cells. (**c**) Box plots show a significantly higher (p < 0.01) colocalization of MGMT with H3K9ac-marked euchromatin domains in drug-treated CP-A and FLO-1 cells and no change in EPC2 cells. Co-localization was assessed through Pearson’s correlation of the pixel intensities of MGMT FISH and H3K9ac staining. Frequency distributions were analyzed using the Kolmogorov-Smirnoff (KS) nonparametric test.

To investigate the interplay between nuclear architecture alterations in neoplastic progression and gene expression induced by vorinostat, we quantified gene position by 3D immuno-FISH and image analysis in normal EPC2, metaplastic CP-A, and malignant FLO-1 esophageal epithelial cells. We selected a candidate set of genes (Supplementary Table S3) on the basis of their reported aberrant expression in Barrett’s Esophagus and esophageal adenocarcinoma. Gene expression assessment by RT-qPCR of this candidate list after vorinostat treatment and normalization to the DMSO control revealed induction of expression of MGMT in all cell lines, with FLO-1 having a 12-fold increase in expression compared to ~2-fold and 5-fold increases observed in CP-A and EPC2, respectively (Supplementary Figure S4). This increase in MGMT expression in FLO-1 after vorinostat treatment was significant when compared to the response of either CP-A or EPC2 (p < 0.0001 for either comparison). MGMT has been shown to be aberrantly silenced by methylation in Barrett’s Esophagus and esophageal adenocarcinoma^31^. We then used immuno-FISH to label the MGMT locus and H3K9ac-marked euchromatin domains in all three cell types, imaged the cells by confocal microscopy, and performed quantitative image analysis on confocal z-stacks to measure the extent of nuclear repositioning of the MGMT gene locus and its colocalization with H3K9ac-marked euchromatin domains. We observed a differential trend in nuclear repositioning as well as H3K9ac-colocalization of MGMT between normal and abnormal cells. Upon drug exposure, the MGMT gene locus moved closer to the geometric nuclear center in CP-A cell nuclei but not in EPC2 cells (Fig. 5a). In FLO-1 cells there was an observed trend toward the center of the nucleus with vorinostat exposure, but this variation did not meet our criterion for statistical significance (p = 0.06). Interestingly, while both the vehicle and drug treated CP-A distributions of relative radial distance (RRD) are obviously non-Gaussian (A = 2.4188, p-value = 3.794e-06 and A = 1.7438, p-value = 0.000172, respectively), the distribution of RRD in FLO-1 becomes Gaussian after treatment with vorinostat. MGMT gene position in DMSO-treated FLO-1 is markedly non-Gaussian (A = 0.94, p = 0.01679), while vorinostat treated cells have RRDs that are normally distributed (A = 0.59, p = 0.1198), with the population as a whole moving toward more median positioning (Fig. 5a). Drug-treated FLO-1 cell nuclei exhibited the greatest heterogeneity in gene repositioning among the 3 cell types (Fig. 5b). The trends observed with FLO-1 cells may be attributed to their inherent aneuploidy at this locus (i.e. the existence of n > 2 alleles of the gene). There is no reason to presuppose *a priori* that all alleles in any given cell would be transcriptionally active and therefore repositioned. Co-localization analysis (Fig. 5c) showed a statistically significant increase (p < 0.01) in MGMT’s spatial association with H3K9ac in drug-treated CP-A and FLO-1 cells, and no change in EPC2 cells. These differential trends between normal and abnormal cells collectively indicate that malignancy-associated changes in 3D nuclear architecture may influence vorinostat-induced increases in gene expression.

## Discussion

Technological advances over the past decade have enabled a better understanding of the three-dimensional spatial architecture of the genome, its alterations with malignancy, and its role in modulating gene expression. However, these aspects have been only sparsely factored into the numerous mechanistic studies undertaken to decipher the tumor-specific action of vorinostat. Hence, we sought to investigate the interplay between the HDAC inhibitor drug vorinostat and the alterations that occur in 3D interphase nuclear architecture of esophageal epithelial cells along a progression spectrum from normal squamous to Barrett’s Esophagus and esophageal adenocarcinoma. In this study, we have demonstrated, quantitatively, that a clinically relevant dose of vorinostat differentially impacts the cytological-scale, 3D interphase nuclear architecture and its heterogeneity in normal, pre-cancerous (metaplastic), and malignant esophageal epithelial cell lines. Upon exposure to the IC50 concentration of the drug, malignant cells preferentially exhibited prominent reductions in the magnitude and heterogeneity of diagnostically relevant morphological parameters such as nuclear volume, nuclear-cytoplasmic ratio, and intra-nuclear chromatin clumpiness relative to pre-cancerous and normal cells. Confocal microscopy revealed that vorinostat mediated a more diffused spatial localization of H3K9ac-marked euchromatin and a slightly increased peripheral localization of H3K9me3-marked constitutive heterochromatin in malignant relative to non-malignant cells. These differential changes after exposure constitute a cancer cell-specific reorganization of the genome at the cytological scale caused by the drug. Consistency in the trends of the observed nuclear structure changes in malignant cells enriched in the G1 and G2 phases of the cell cycle showed that the phenomena are not tied to potential drug-induced cell cycle blocks or senescence. Further, we showed that drug-induced expression of the MGMT gene was associated with its increased colocalization with H3K9ac-marked euchromatin domains in pre-cancerous and malignant cells, but not in normal cells. Further there was a preferential inward nuclear relocation of the gene in metaplastic cells. In cancer cells, there was not a significant change in gene localization, but there was a redistribution of gene positions in these cells. These results point to a functional role for 3D nuclear architecture in vorinostat’s tumor-selective action on esophageal epithelial cells. They also represent the first demonstration of the utility of 3D nuclear morphometry as a quantitative read-out to assess cellular response to vorinostat treatment in adenocarcinoma.

The optical-CT based 3D imaging and morphometry methods used in this study enable a close correlation between hematoxylin-stain based morphological observations of cell nuclei commonly employed in clinics by pathologists and fluorescence microscopy observations made by the research community. The use of optical CT to volumetrically image fixed hematoxylin-stained cells at sub-micron, isotropic spatial resolution provides the ability to rapidly and accurately assess 3D nuclear structure and its heterogeneity at the cytological scale. The isotropy in spatial resolution, inherent to this technology, precludes the need for image interpolation procedures that artificially synthesize 3D confocal microscopy images with isotropic voxel dimensions. The power to generate accurate, quantitative 3D nuclear morphometry pre- and post-treatment allows generation of robust morphological signatures. This approach holds promise for clinical application in monitoring or predicting therapeutic response to vorinostat or other drug treatment. It is not unreasonable to suggest that clinical utility of vorinostat in combination therapy as a sensitizer is due in part to the observable changes in nuclear structure that it induces. By following patients, either through serial biopsy or circulating tumor cells, and analyzing the nuclear structure of the cell population, we could not only determine what proportion of cells are responding to the vorinostat but whether those cells represent a malignant, hyperplastic or normal component of the tumor mass. This type of single cell analysis would be particularly useful in contexts where the ability to detect the cells of interest is confounded by the presence of vast numbers of normal or off-target cells. The measurement of drug-induced changes in higher-order genome architecture by quantitative 3D immuno-FISH as performed in this study could also be useful tool to evaluate therapeutic response.

Our study suggests possible efficacy of vorinostat as a single therapeutic agent to treat Barrett’s Esophagus and esophageal adenocarcinoma. Although vorinostat has been shown to be effective for hematological malignancies and is in numerous trials for synergistic therapeutic interventions, little is known about the effect of vorinostat on esophageal adenocarcinoma (EA). The sub-micromolar IC50 drug concentration for malignant esophageal epithelial cells and the large IC50 differential relative to metaplastic and normal squamous cells point to vorinostat’s specificity for transformed cells of this cancer type. The two-fold difference in IC50 values between metaplastic and normal squamous cells suggests that the drug could potentially be of benefit for Barrett’s Esophagus patients. It would be interesting to evaluate whether vorinstat treatment could block progression of esophageal adenocarcinoma at a pre-cancerous stage by normalizing Barrett’s Esophagus cells. Our studies also confirmed the time- and dose-dependent inhibitory activity of vorinostat. Similar trends have been reported for other FDA-approved HDAC inhibitors such as panabinostat^32^.

Our results from this neoplastic progression model system provide novel insight into the mechanism of action of vorinostat by demonstrating state-specific effects of the drug on higher-order 3D genome architecture of epithelial cells in their progression from normal to malignant states. The correlation between the 3D nuclear architecture changes in vorinostat-treated malignant cells and their reduced viability relative to treated precancerous and normal cells raises the possibility that malignant cells may actually require aberrant genome architectures for their survival and function. While histone acetylation is often thought of as a mark of “open” chromatin, and therefore HDAC inhibition with vorinostat would be expected to increase nuclear volume through chromatin decompaction, our results suggest a more nuanced picture. We postulate that the decrease in nuclear volume that we see after vorinostat exposure is a consequence of the disruption of the dynamics of both histone acetylation at multiple lysines, as well as DNA binding proteins that results in a more normal nuclear volume in metaplastic and cancer cells. Since these cells have significantly larger nuclear volumes to begin with, these alterations of chromatin structure leads to a decrease in nuclear volume resulting in nuclei more similar in size to those of normal cells. A useful future study to gain a better understanding of the variation in 3D nuclear structure would be to image cells at regular intervals post-vorinostat treatment and annotate their nuclear morphology. Such a study would provide potentially useful supplementary information on the changes we observed at 48 hours.

The difference between normal and abnormal cells in MGMT gene position and its colocalization with euchromatin domains suggest a connection between gene regulation, nuclear position, and cancer-related 3D nuclear architecture. The epigenetic disruption of 3D nuclear architecture caused by vorinostat in abnormal cells may represent a biophysical basis for the drug’s tumor-selective mechanism of action. Our results and perspective on vorinostat’s mechanism of action aligns with the idea that intrinsic chromatin structure differences between normal and malignant cells underlie the cancer specificity of HDAC inhibitors generally^33^. This however does not preclude the possibility of other mechanisms such as differential DNA damage repair capabilities^11^ or the suppression of molecules that neutralize reactive oxygen species^34^. Other possible mechanisms consistent with our results on differential nuclear re-organization could be the disruption of ATP levels and pH buffering capacity in drug-treated cancer cells. McBrian *et al*.^35^ have postulated that drug-treated cancer cells could suffer misregulation of intracellular pH due to hyperacetylated chromatin. We theorize that this misregulation may extend to extracellular pH and intracellular ATP levels as well since esophageal adenocarcinoma cells are known to reside in a lower pH environment and consume less oxygen relative to normal squamous cells^36^. Sensor technologies are available to validate these hypotheses in future experiments^37^.

The data we report on the differential trends between drug-treated normal and abnormal cells in nuclear positioning and H3K9ac-colocalization of MGMT raise intriguing questions about the functional significance of nuclear position. The consistency of co-localization of the gene locus with H3K9ac-marked euchromatin domains in drug-treated abnormal cells indicates that the local chromatin environment surrounding a gene could exert a stronger influence on its expression level than its nuclear position. The result thus reaffirms prior reports about local chromatin environments potentially taking precedence over a gene’s spatial location within the nucleus in epigenetically modulating its expression^38^. It would be of value to conduct immuno-FISH or live cell experiments with other genes to determine whether this finding is generalizable.

We recognize that some of our results contrast with previously published findings. The observed reduction in nuclear volume we report accompanying the increase in chromatin decompaction in vorinostat-teated malignant esophageal epithelial cells is at odds with prior studies that report the opposite trend in lung and colon cancer cells in response to trichostatin A^24^. This may be a drug-specific or cell line dependent phenomenon, as may the lack of cell cycle blocks seen in the drug-treated FLO-1 cells. Among the other observations that warrant additional investigation are the increases measured in the nuclear-cytoplasmic ratio and intra-nuclear clumpiness in drug-treated interphase normal squamous cells. The decrease in lamin B1 expression but not lamin B2 and lamin A/C also requires further analysis. Recent studies have reported a reduction in lamin B1 levels in senescent cells upon DNA damage^39^. Although this aspect does not appear to be directly germane to our study, ChIP-seq experiments could provide insight into the functional implication of lamin B1 loss on the 3D genome architecture of cancer cells. Another beneficial direction for future investigation would be to assess the temporal changes in 3D nuclear architecture in normal and abnormal cells after withdrawal of vorinostat. Such a study would provide insights into the extent to which the reversible nature of epigenetic drug action percolates through length scales in genome architecture. Lastly, we recognize the limitations imposed by the use of immortalized cell lines cultured in 2D conditions to model neoplastic progression in Barrett’s Esophagus. Cells cultured in 3D matrices or retrieved from mouse models that spontaneously recapitulate the normal progression to esophageal adenocarcinoma would provide greater translational relevance and facilitate investigations into the biophysical impact of the microenvironment on genome architecture. Nevertheless, our study emphasizes the need to incorporate the spatiotemporal behavior of higher-order chromatin structure and its malignancy-associated alterations into studies that seek to understand the utility of vorinostat in treating solid tumors.

## Methods

### Cell culture and reagents

Our choice of cell lines was motivated by their representation of neoplastic progression from normal squamous to esophageal adenocarcinoma (EA) through a metaplastic stage called Barrett’s Esophagus (BE). Aberrant expression of HDAC 1 and 2 has been previously reported in BE and EA^40^,^41^. hTERT-transformed “normal” squamous (EPC2), metaplastic Barrett’s Esophagus (CP-A), and esophageal adenocarcinoma (FLO-1) cell lines were cultured per ATCC-prescribed protocol as monolayers in T-25 flasks at 37 °C and 5% CO_2_. Cells were grown to approximately 85% confluence prior to the dosing experiments. Vorinostat (suberoylanilide hydroxamic acid) was dissolved in dimethyl sulfoxide as a 100 mM stock solution prior to storage at −20 °C.

### Vorinostat treatment

EPC2, CP-A, and FLO-1 cells were seeded in 96-well plates at 3000 cells per well. After 24 hours, a 10 point, 3-fold serial dilution starting at 100 μM was added to the plates. Cell viability was measured using the CellTiter Glo assay (Promega, Madison, WI). Viability data was normalized to cells alone and then to cells treated with vehicle alone. The resulting data (n = 6 for each dose) were analyzed by using the Dose-response-inhibition, variable slope function in Prism 6 (Graphpad Software, San Diego, CA) and the concentration corresponding to 50% cell viability (the IC50) was determined. The program fits the sigmoidal equation Y = Bottom + (Top-Bottom)/(1 + 10 ^ ((LogIC50-X)*HillSlope)). For all additional experiments, cells were seeded 24 hours prior to treatment for 48 hours with 0.94 μM vorinostat and immediately harvested after treatment for analysis. DMSO was used as the vehicle control.

### Cell cycle analysis

Cells for each condition (untreated, DMSO-treated, and vorinostat-treated) were stained with 10 μL/mL Hoechst 33342 live cell DNA dye, incubated for 10 minutes, then resuspended in PBS. Stained cells were sorted into G1 and G2 phases by fluorescence activated cell sorting (FACS).

### Reverse transcription quantitative PCR (RT-qPCR)

Total RNA was extracted from cells using the Qiagen RNeasy mini kit (Qiagen, Valencia, CA) per manufacturer’s protocol. cDNA was synthesized using the iScript Reverse Transcription Supermix (Bio-Rad, Hercules, CA). Primers for target genes (MLH1, MGMT, CDKN2A (p16), CDKN1A (p21), CDH1, CDX1, CDX2, TFF3, AKAP12, and EZH2) were procured from Qiagen, the normalizing control gene β-actin was custom designed (Supporting Table 3). RT-qPCR was performed using SYBR-Green Master Mix (Life Technologies, Grand Island, NY) on the Step One Plus platform (Applied Biosystems, Foster City, CA). Reactions were run as follows: 1 cycle at 95 °C for 2 minutes followed by 40 cycles of the sequence: 95 °C (15 seconds), 60 °C (1 minute), and 72 °C (30 seconds). The acquired data was analyzed using the Pfaffl method^42^ to obtain the expression levels of target genes relative to the control gene.

### Immunoblotting

Cells were lysed using RIPA buffer containing 1% sodium orthovanadate and protease inhibitors, transferred to 2 mL Eppendorf tubes, incubated for 30 minutes at 4 °C while rotating, and centrifuged. Protein concentrations were quantified using the Pierce BCA protein assay kit (23225, Thermo Fisher, Waltham, MA). Extracted proteins were loaded into 30 μL wells of 4–15% Immobilion Protean gels (446–1083, Bio-Rad, Hercules, CA), electrophoresed then transferred to Immobilion PVDF membranes. Membranes were blocked for one hour in Licor blocking buffer (927–40,000, LI-COR, Lincoln, NE), incubated overnight at 4 °C with the appropriate primary antibodies, washed four times with 0.1% Tween20 in PBS (PBST) and finally incubated for one hour with the appropriate secondary antibodies (Licor IRDye 680/800). Experiments were performed in triplicate for each condition for the following proteins: H3K9ac (Millipore, #06–942), H3K9me3 (Abcam, Cambridge, MA, ab8898), H3K27me3 (Millipore, #07–449), lamin A/C (Cell Signaling Technology, Danvers, MA, #2032), lamin B1 (Abcam, ab16048), and lamin B2 (Abcam, ab8983). β-actin (Sigma Aldrich, A2103) was used as the loading control. The blots were imaged and quantified using the Odyssey platform and software (LI-COR).

### Immunofluorescence

Cells were cultured on 1.5 thickness rectangular cover glasses. Prior to immunolabeling, cells were fixed for 5 minutes with freshly prepared 2% formaldehyde, permeabilized with Karsenti’s buffer (0.5% Triton X-100, 80 mM PIPES, 1.0 mM MgSO4, 5.0 mM EGTA, pH 7.0) for 2 minutes, fixed again with 2% formadehyde for 2 minutes, rinsed 3 times with PBST, then blocked with 1% bovine serum albumin (BSA) and 5% normal goat serum in PBST for 30 minutes. Cells were subsequently immunolabeled by incubation with primary antibodies diluted in PBST with 1% BSA for either 2 hours at room temperature or overnight at 4 °C, then incubated 1–2 hours in the appropriate AlexaFluor™ conjugated secondary antibody (Life Technologies) diluted in PBST with 1% BSA. Immunolabeled cell nuclei were finally counter-stained with 10 μg/mL DAPI for 20 minutes at room temperature. Cells were rinsed 3 times in PBST after each labeling step and an additional 2 times in PBS after the final step. Finally, the cover glasses were mounted onto a glass slide in phosphate buffered 90% glycerol and sealed with clear nail polish. Spatial localization was assessed for the following proteins: lamin A/C (Santa Cruz, sc-7292), fibrillarin (Abcam, ab4566), H3K9ac (Millipore, #06–942), H3K9me3 (Abcam, ab8898, and Novus Biologicals, NBP1-30141), and H3K27me3 (Abcam, ab6002 and Millipore, #07–449). Stained cells were imaged by laser scanning confocal using a 60×, 1.2 NA, water-immersion lens. All experiments were performed in triplicate. Image acquisition settings were consistent across cell lines and treatments. Images were post-processed using the Nikon NIS Elements software package.

### Single-cell optical CT imaging and 3D nuclear morphometry

The Cell-CT™ platform (VisionGate, Phoenix, AZ) was used to perform rapid single-cell optical CT imaging. This absorption-based imaging modality generates 3D images of fixed and stained, individual cells at isotropic sub-micron spatial resolution^30^. In contrast to the conventional approach of generating volumetric images by stacking 2D images (e.g. widefield or confocal microscopy), 3D images in optical CT are generated by tomographic reconstruction from 500 angular pseudo-projections, of a fixed, stained single cell inside a rotating glass capillary. Supplementary Figure S5 demonstrates the morphological distortion induced by confocal microscopy due to its inferior spatial resolution along the optical axis, and how this problem is overcome by the isotropic resolution inherent to optical cell CT imaging. Protocols for sample preparation and image acquisition were previously described^43^. Briefly, trypsinized cells were fixed for 1 hour with CytoLyt (Cytyc, Marlborough, MA). Fixed cells were stained with 6.25% w/w hematoxylin, embedded in an optical gel (Smartgel, Nye, Fairhaven, MA) and loaded into a 100 μL glass syringe. Interphase cells were tomographically imaged using the Cell-CT™, and the reconstructed 3D images were subjected to automated morphological analysis using Matlab (version 2011a, Mathworks, Natick, MA) to quantify cellular features (defined in Supplementary Table 1). Two hundred cells were analyzed per condition (control, vehicle-treated, and drug-treated) for unsynchronized EPC2, CP-A, and FLO-1 cells. In addition, 100 control and drug-treated FLO-1 cells were imaged after sorting by FACS for enrichment in the G1 and G2 phases of the cell cycle.

### 3D Fluorescence *In Situ* Hybridization (FISH) and image analysis

TAMARA-labeled FISH probes (RP11-809J17 and RP11-779G23) were procured from Empire Genomics (Rosewell Park, NY). Cells were grown in chambered cover glasses, and subject to 3D immuno-FISH^44^ to concurrently label the nuclear position of the target gene and assess its colocalization with euchromatic domains represented by the histone mark H3K9ac. Adherent cells were fixed in 4% formaldehyde at room temperature for 10 minutes, washed 3 times with PBS, permeabilized with 0.5% Triton X- 100 in PBS for 15 minutes, blocked at room temperature for 10 minutes with 4% BSA in PBS, incubated with primary antibody at 37 °C for 1 hour, washed 2 times (5 minutes each) with PBST, incubated with AlexaFluor 488 conjugated goat anti-rabbit IgG secondary antibody (A-11034, Life Technologies) for 1 hour, and washed twice with PBST. To prepare for FISH, the cells were then post-fixed in 1% formaldehyde for 10 minutes and permeabilized again in 0.5% Triton X-100 in PBS for 5 minutes. Slides were then incubated in 0.1M HCl for 7 to 10 minutes at room temperature, washed twice (5 minutes each) with 2X salt sodium citrate (SSC), treated with RNase (100 μg/mL in SSC) for 1 hour at 37 °C and allowed to equilibrate in 50% formamide/2X SSC for at least 2 hours. After equilibration, slides were denatured at 85 °C for 3 minutes with hybridization mixture (2 μL of FISH probe, 8 μL of hybridization buffer, and 0.5 μL of Cot1 DNA) and incubated overnight at 37 °C in a humidified container. On the following day, cells were washed three times at 37 °C in 2X SSC for 5 minutes per wash. This was followed by washing the slides three times for 5 minutes per wash in 0.1X SSC at 60 °C. The cells were stained with DAPI prior to mounting.

Three-D image stacks of labeled cells were acquired at an axial step-size of 0.25 μm using a 60×, 1.4 NA oil-immersion lens and laser scanning confocal imaging. Image acquisition settings were consistent across experiments. Matlab software was used to quantify gene localization in the acquired 3D image data. The relative radial distance (RRD) metric (adapted from^45^) was applied to determine the position of the FISH signal relative to the nuclear periphery (0 implies location at the periphery, and 1 implies location at the nuclear center). The Pearson’s coefficient was calculated to measure the extent of H3K9ac co-localization with the FISH signal.

### Statistical analysis

Statistical analyses were performed using Prism 6 software (Graphpad Software, San Diego, CA). Differences between treated cell lines and DMSO controls were assessed by Mann-Whitney due to the non-Gaussian nature of the data. Additionally, the Kolmogorov-Smirnoff (KS) was applied to compare the distributions of parameters, and the adjusted p-values are reported. P-values below 0.05 were considered to be significant. Normality was assessed by Anderson-Darling Normality test using ad.test from the nortest package in R.

## Additional Information

**How to cite this article**: Nandakumar, V. *et al*. Vorinostat differentially alters 3D nuclear structure of cancer and non-cancerous esophageal cells. *Sci. Rep*. **6**, 30593; doi: 10.1038/srep30593 (2016).

## Supplementary Material

Supplementary Information

## Figures and Tables

**Table 1:** Statistics of diagnostically relevant morphometry measurements derived from 3D optical CT images of unsynchronized hTERT-transformed squamous (EPC2), metaplastic (CP-A), and adenocarcinoma (FLO-1) esophageal epithelial cells treated with the vehicle control (DMSO) and vorinostat (vstat).

Morphological descriptor	Treatment condition	Quantitation (mean ± standard error of the mean)		
		EPC2-hTERT (Normal)	CP-A (Metaplasia)	FLO-1 (Cancer)
Nuclear volume (μm^3^)	DMSO	356.8 ± 10.3	953.3 ± 46.8	672.7 ± 22.9
	vstat	381.6 ± 10.4	896 ± 42.6	553.2 ± 20.5
Nuclear-cytoplasmic ratio	DMSO	0.19 ± 0.01	0.52 ± 0.02	0.65±0.02
	vstat	0.22 ± .01	0.26 ± 0.01	0.51 ± 0.01
Nuclear shape concavity (%)	DMSO	6.3 ± 1.1	12.7 ± 1.3	11 ± 0.8
	vstat	8.2 ± 1.2	27.1 ± 2.4	9.5 ± 0.8
Number of dense clumps in nucleus	DMSO	3.9 ± 0.18	6.1 ± 0.24	6.1 ± 0.23
	vstat	4.5 ± 0.17	5.8 ± 0.22	5 ± 0.17

**Table 2:** Statistics of diagnostically relevant morphometric measurements derived from 3D optical CT images of untreated and vorinostat (vstat)-treated adenocarcinoma (FLO-1) esophageal epithelial cells in G1 and G2 phases of the cell cycle.

Morphological descriptor	Treatment condition	Quantitation (mean ± standard error of the mean)	
		FLO-1 G1	FLO-1 G2
Nuclear volume (μm^3^)	untreated	516.3 ± 28.1	503 ± 19.5
	vstat	343 ± 17.5	407 ± 16.5
Nuclear-cytoplasmic ratio	untreated	0.45 ± 0.01	0.51 ± 0.02
	vstat	0.39 ± 0.02	0.39 ± 0.01
Nuclear shape concavity (%)	untreated	11.1 ± 1.2	15 ± 1.7
	vstat	12.6 ± 1.7	13.6 ± 1.9
Number of dense clumps in nucleus	untreated	8.5 ± 0.5	6.1 ± 0.3
	vstat	5.1 ± 0.3	4.2 ± 0.2
