# Supplementary material for: Vorinostat differentially alters 3D nuclear structure of cancer and non-cancerous esophageal cells
Source: Sci Rep. 2016 Aug 9;6:30593. doi: 10.1038/srep30593 (PMC4977554; doi:10.1038/srep30593)
Supplement: Supplementary Information [file srep30593-s1.pdf]

Vorinostat differentially alters 3D nuclear structure of cancer and non-cancerous esophageal cells

Vivek Nandakumar, Nanna Hansen, Honor L. Glenn, Jessica Han, Stephanie Helland, Kathryn Hernandez, Patti Senechal, Roger H. Johnson, Kimberly J. Bussey, and Deirdre R. Meldrum

Table S1: Definition of morphological parameters measured from 3D images

Morphological descriptor	Description
Nuclear volume (μm^3)	(volume of 1 voxel in image) × (total number of voxels in segmented 3D nuclear mask)
Nuclear-cytoplasmic ratio	Ratio of nuclear volume to volume of cytoplasm, where the latter is defined as the difference between cell volume and nuclear volume
Nuclear shape concavity index	$\left(\frac{Volume\ of\ smallest\ enclosing\ convex\ shape - nuclear\ volume}{Volume\ of\ smallest\ enclosing\ convex\ shape}\right) \times 100$
Number of dense clumps in nucleus	Calculated by segmenting dense clumps in 3D image based on their high image intensity. The minimum clump size was empirically set to be 100 voxels

Table S2: Summary of cell cycle analysis by flow cytometry (N=3). The average % value of each population is listed.

Cell type	Condition	Phase in cell cycle (% mean proportion + standard deviation)				
		**sub-G1**	**G1**	**S**	**G2-M**	**other**
Normal squamous (EPC2)	Control	4.7 ± 2.1	76 ± 5.6	11 ± 2	6.7 ± 3	1
	DMSO	35 ± 5	55.3 ± 8.5	5.7 ± 4	1.3 ± 0.6	2 ± 2.6
	vorinostat	25.6 ± 5.5	66.3 ± 7.7	4.3 ± 2.1	1.3 ± .6	1
Metaplasia (CP-A)	Control	9.3 ± 5.7	34.7 ± 1.1	14 ± 3	30 ± 8	11 ± 2.6
	DMSO	13 ± 8	40.7 ± 2.5	13.3 ± 1.2	24.3 ± 10	7 ± 1.7
	vorinostat	37.7 ± 7.1	35.3 ± 3.1	12.3 ± 4	14 ± 2	4
Adenocarcinoma (FLO-1)	Control	34.7 ± 14.2	32.7 ± 7.4	14.3 ± 2.1	9 ± 4.6	8.7 ± 4.9
	DMSO	23.7 ± 11	48.7 ± 5.7	11 ± 1	10 ± 4.4	5.7 ± 2.3
	vorinostat	25.3 ± 6	47.3 ± 2.5	11.7 ± 1.5	9 ± 1.7	5.7 ± 2.5

Table S3: Information about primers used for qRT-PCR

Gene name	Manufacturer	Product information
CDKN2A (p16)	SA Bioscience/Qiagen	PPH00207C
TFF3	SA Bioscience/Qiagen	PPH00449A
CDX1	SA Bioscience/Qiagen	PPH01470F
CDX2	SA Bioscience/Qiagen	PPH13618B
MGMT	SA Bioscience/Qiagen	PPH01519E
AKAP12	SA Bioscience/Qiagen	PPH06033A
CDKN1A	SA Bioscience/Qiagen	PPH00211E
MLH1	SA Bioscience/Qiagen	PPH00196E
EZH2	SA Bioscience/Qiagen	PPH02880A
CDH1	SA Bioscience/Qiagen	PPH00135E
β-actin	custom	

Figure S1:

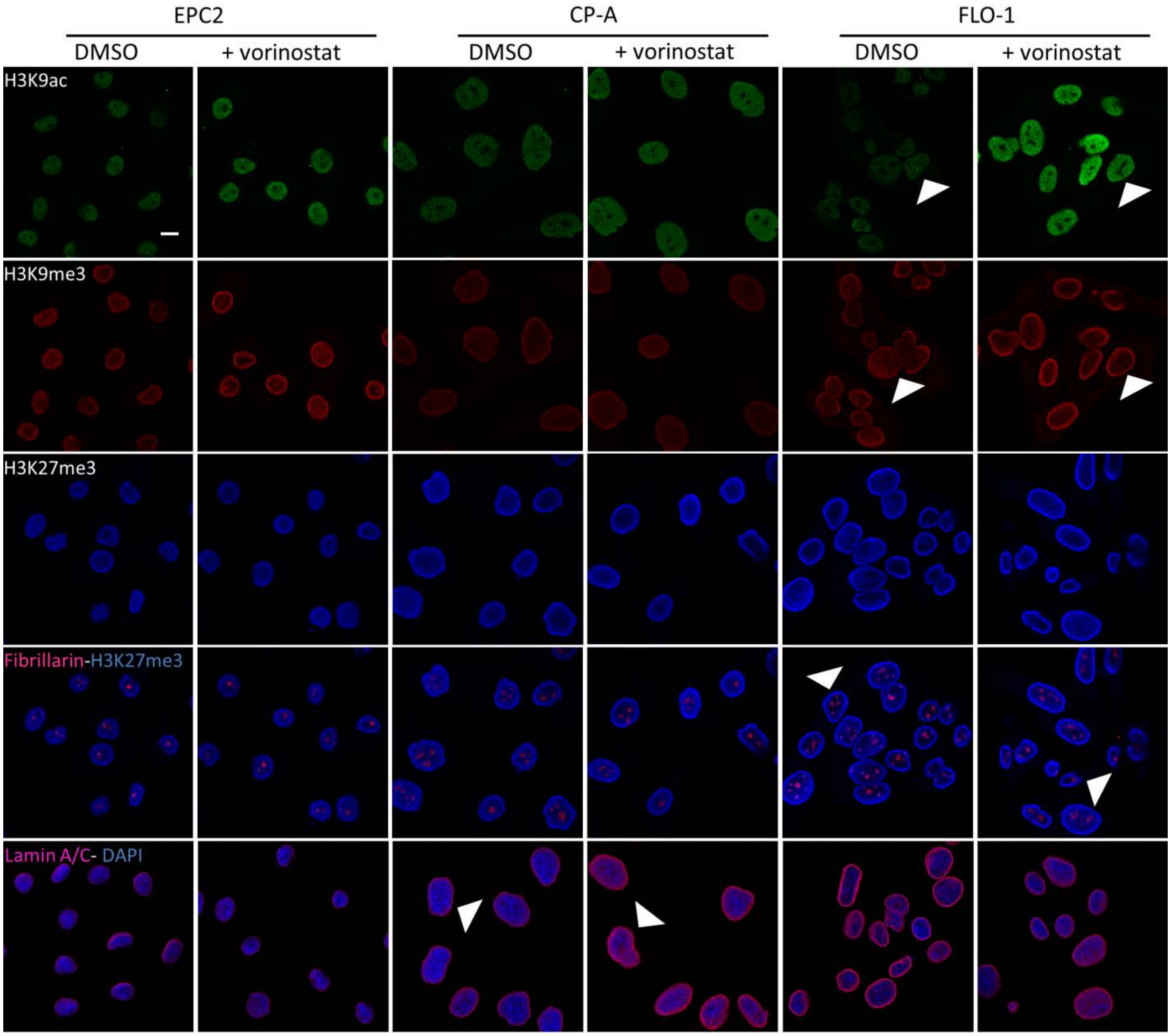

Figure S1: Immunofluorescence confocal microscopy of adhered cells validates morphological observations made using single-cell optical CT. Vorinostat treatment preferentially decreased chromatin compaction in FLO-1 esophageal adenocarcinoma cells and increased nuclear shape concavity in metaplastic CP-A cells (see arrowheads). Representative confocal micrographs of immunolabeled cells illustrate the significant increase in euchromatin mark H3K9ac, peripheral marginalization of constitutive heterochromatin mark H3K9me3, and a decrease in nucleolar protein Fibrillarin in a subset of FLO-1 cancer cells, and an increased nuclear surface undulations (marked by lamin A/C) in metaplastic CP-A cells upon treatment with vorinostat. No significant change was observed in the localization of facultative heterochromatin mark H3K27me3.Scalebar = 10 microns.

Figure S2:

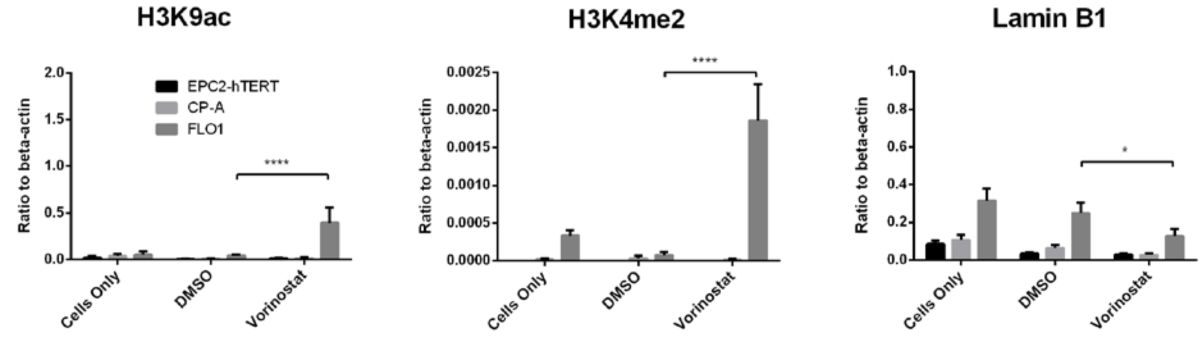

Figure S2: Treatment with vorinostat preferentially altered the expression of proteins that underlie nuclear shape and higher order chromatin structure. Bar graphs derived from immunoblotting experiments illustrate increased levels of active histone marks H3K9ac and H3K4me2, and decreased Lamin B1 expression in FLO-1 esophageal adenocarcinoma cells relative to metaplastic CP-A and normal squamous EPC2 cells upon treatment with vorinostat. The number of *s indicates the statistical significance for the comparison with DMSO (* = p <0.05, ** = p<0.01, *** = p<0.001, **** = p<0.0001). Graphs were derived from the analysis of three technical and three biological replicates per condition per cell line. The reported expression levels of target proteins after normalization with β-actin.

Figure S3:

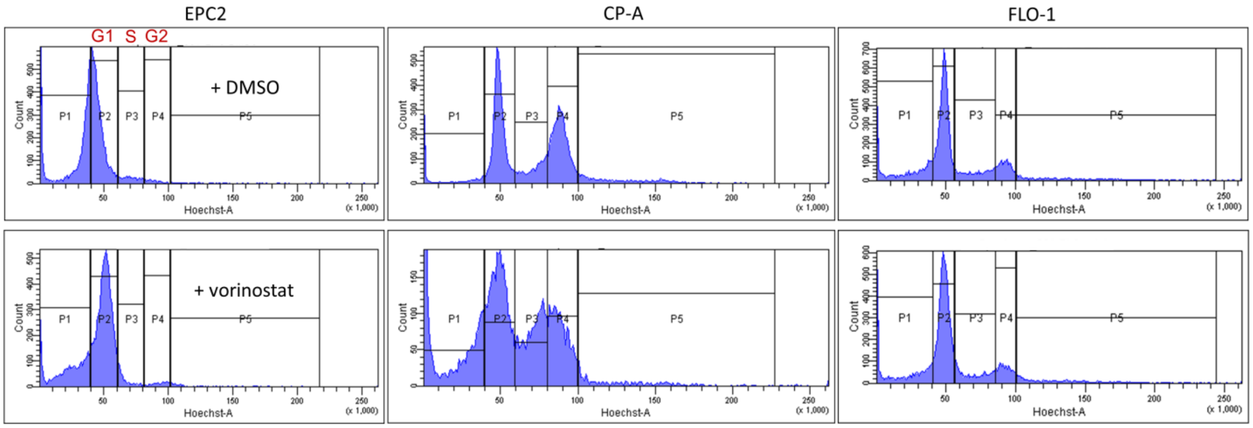

Figure S3: Vorinostat treatment minimally alters cell cycle distributions in FLO-1 cancer cells relative to vehicle control (DMSO). Graphical representation of fluorescence activated cell sorting (FACS) data on Hoescht stained cells shows a minimal change between DMSO- and vorinostat-treated FLO-1 cells. In contrast, drug-treated normal squamous EPC2 cells appear to undergo a cell cycle shift towards the G1 and sub-G1 phases while metaplastic CP-A cells additionally exhibit a shortened S-phase and increase in G2/M.

Figure S4

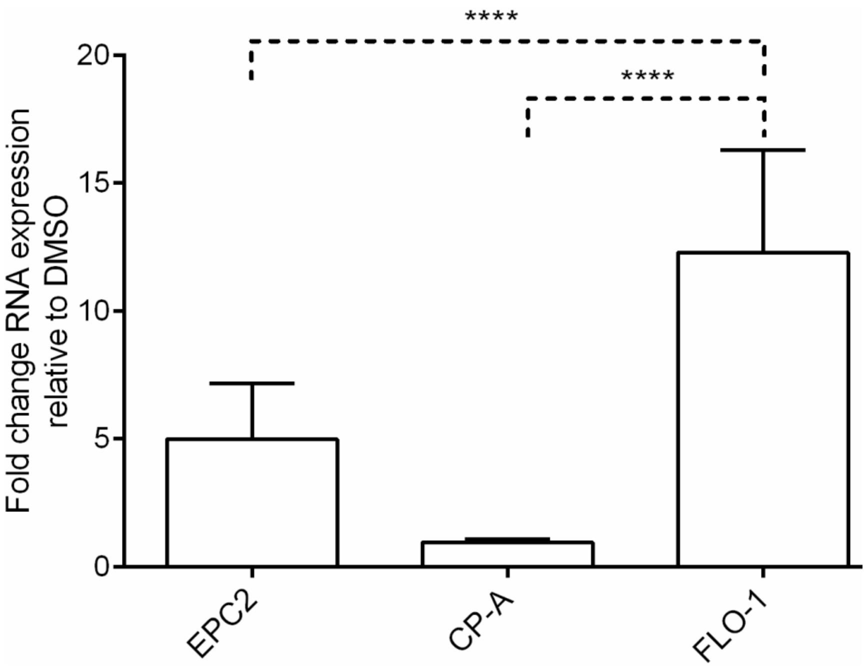

Figure S4: Vorinostat exposure causes a differential mRNA expression of MGMT in normal and abnormal cells. RT-PCR analysis revealed a very significant induction in the expression of MGMT in FLO-1 cells compared to CP-A and EPC-2 cells. Plotted data was generated from 3 biological and technical replicates. * indicates the extent of statistical significance in the comparison (ns = not significant, * = p<0.05, ** = p<0.01, *** = p<0.001, **** = p<0.0001).

Figure S5:

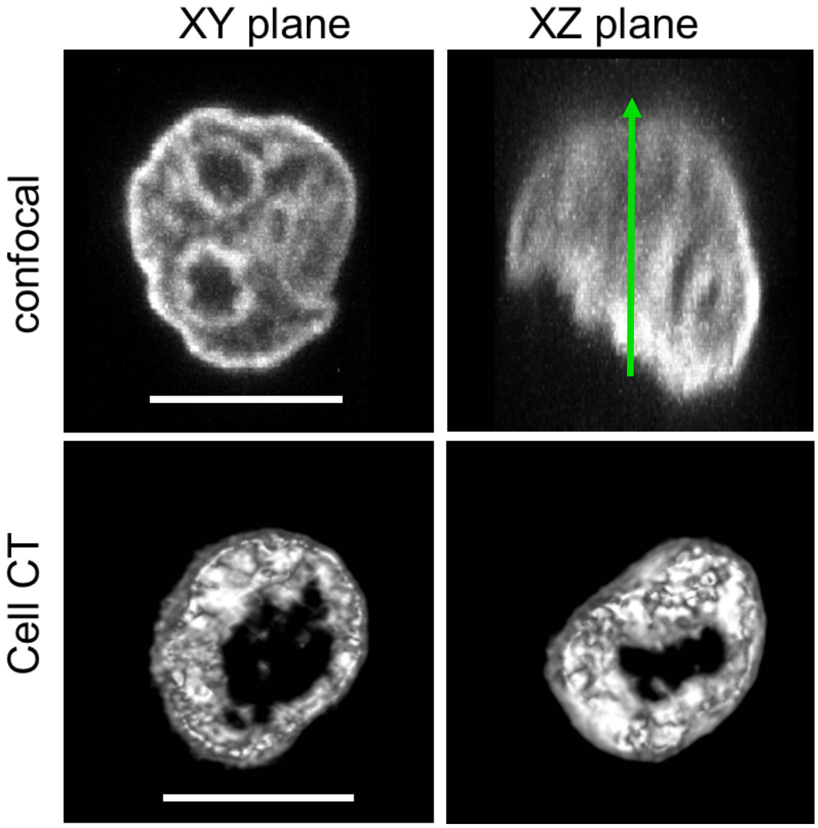

Figure S5: Nuclei of FLO-1 cells imaged by confocal (top) or Cell CT (bottom). Left and right panels are two, orthogonal, 1 µm optical sections through the nucleus in the xy or xz planes. Distortion of the nuclear shape is observed in the confocal image due to the inferior spatial resolution along the optical axis (z axis). The optical cell CT image features isotropic resolution with no such distortions. The green arrow represents the optical axis of the imaging system. Scale bar: 10 µm.
